# Strategies for dispersion of cariogenic biofilms: applications and mechanisms

**DOI:** 10.3389/fmicb.2022.981203

**Published:** 2022-09-02

**Authors:** Rourong Chen, Minquan Du, Chang Liu

**Affiliations:** The State Key Laboratory Breeding Base of Basic Science of Stomatology (Hubei-MOST), Key Laboratory of Oral Biomedicine Ministry of Education, School and Hospital of Stomatology, Wuhan University, Wuhan, China

**Keywords:** dispersion, eradication, disruption, cariogenic biofilms, dental plaque, *Streptococcus mutans*

## Abstract

Bacteria residing within biofilms are more resistant to drugs than planktonic bacteria. They can thus play a significant role in the onset of chronic infections. Dispersion of biofilms is a promising avenue for the treatment of biofilm-associated diseases, such as dental caries. In this review, we summarize strategies for dispersion of cariogenic biofilms, including biofilm environment, signaling pathways, biological therapies, and nanovehicle-based adjuvant strategies. The mechanisms behind these strategies have been discussed from the components of oral biofilm. In the future, these strategies may provide great opportunities for the clinical treatment of dental diseases.

**Graphical Abstract:**
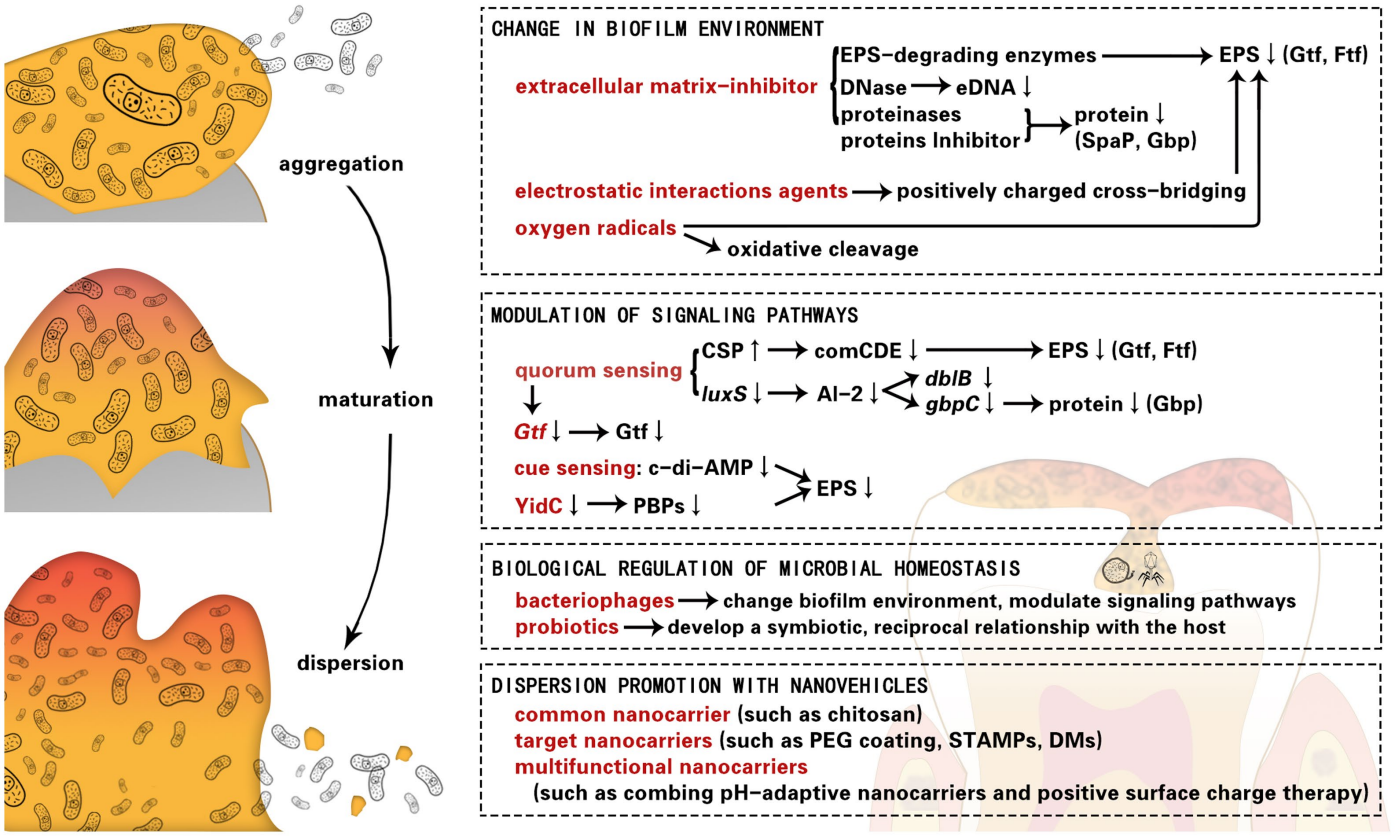

## Introduction

Dental caries is a common oral disease that is mainly caused by cariogenic biofilm. Cariogenic biofilms constantly form in the oral cavity. Besides mechanical cleaning, auxiliary chemical methods are necessary to control their spread (Pratten et al., 1998).

For better physical settlement, microorganisms produce, and wrap themselves in a matrix that acts like a “protective scaffold” (Jamal et al., 2018). As an architectural colony, the microbial ecosystem of caries is an ordered and spatial community (Kim and Koo, 2020), which offers opportunities for close relationships and high mutation frequency to virulence genes. In addition, as active and complex organizations, cariogenic biofilms colonize competitive niches and are resistant to stressful environments. Cariogenic biofilms restrict and sequestrate the penetration of chemicals through the matrix (Sims et al., 2020). Therefore, it is not surprising that bacteria in the biofilm state are more tolerant to various antibiotics, and thus, are more difficult to control than bacteria in the planktonic state (Davies, 2003). With long-term applications, antibiofilm drugs may not only induce resistance of cariogenic bacteria but also disrupt healthy microbiota, resulting in the limitation of existing antimicrobial therapies (Perez-Diaz et al., 2015).

Several studies have focused on the inhibition of biofilms and most present agents can inhibit biofilm-forming bacteria without eradicating the mature biofilm. Due to the short effect time of inhibitors, they cannot control the biofilm well. Therefore, to some extent, dispersion and eradication of mature biofilms are very important for biofilm control.

Most strategies for cariogenic biofilm dispersion are restricted to a particular approach; however, each approach has advantages and disadvantages. It is important to reinforce the concept of co-administration of different strategies. In this review, we summarize the applications and mechanisms of the strategies for dispersion of cariogenic biofilms, including changing the micro-environment, modulating signaling molecules, and so on. In addition, we explore a few novel biological and nanovehicle-based strategies, which have the potential to be combined with traditional approaches or strengthen the effects of cariogenic biofilm dispersion.

## The biofilm lifestyle and dispersion

The life cycle of cariogenic biofilms has already been studied thoroughly. The development of biofilms is generally considered to be a different stage of a cyclic process. During the infection, biofilm formation is initiated by the aggregation of planktonic cells. In the biofilm, single bacterial cells are protected against the immune system and antimicrobial agents (Serra and Hengge, 2014). The concentration gradient of oxygen, nutrient resources and waste products become steepening. These stress factors of the different micro-environment may activate the starvation mechanisms and accumulation of molecules to induce dispersion (Nguyen et al., 2011). The life cycle of a biofilm is finalized with the cells escaping via dispersion to new sites for colonization. The biofilm releases the bacterial cells and allows them to recolonize at other sites (Koo et al., 2017). Evacuation of bacteria leave behind voids in the center of mature biofilm (Rumbaugh and Sauer, 2020). Although self-disassembly can result in infection and bacteremia (Fleming and Rumbaugh, 2018), the dispersed bacteria and biofilm with center voids become much more sensitive to antimicrobial agents.

Therefore, in the final stage of biofilm development, dispersion provides a great opportunity for us to remove biofilm unaffectedly (Lin et al., 2022). It is possible to create an environment that is experienced by bacteria in biofilms during the terminal stages (such as by mediating extracellular signaling molecules, nutrient resources, and oxygen) to induce biofilm degradation and diffusion. Taking advantage of the metabolites or enzymes of microorganisms would be a gentle and specific approach that would not affect the development of dysbiosis or the balance of the beneficial oral microbiome (Pleszczynska et al., 2015). Therefore, biofilm life cycles can be exploited in effective biofilm dispersion strategies.

## Change in biofilm environment

### Extracellular matrix-inhibitor

Degradation of the matrix is an effective strategy for the “physical collapse” of the biofilms (Rainey et al., 2019). As mentioned before, the extracellular matrix is a shield for the biofilm residents, which not only provides structural protection to encase the community but also gain nutrients for metabolic utilization. Generally, these matrices comprise extracellular polymeric substances (EPS), extracellular DNA (eDNA), proteins, and lipids (Petersen et al., 2005; Jakubovics and Burgess, 2015).

#### Extracellular polymeric substance-degrading enzymes and inhibitor

Extracellular polymeric substances is one of the most important components in cariogenic biofilm matrices and includes glucans, and fructans. The polysaccharides in EPS promote bacterial aggregation and mediate biofilm adhesion (Lynch et al., 2007), which aids in avoiding a collapse of the biofilm architecture (Liljemark and Bloomquist, 1996). EPS are synthesized by extracellular enzymes of oral bacteria (Townsend-Lawman and Bleiweis, 1991; Vacca Smith and Bowen, 2000), such as glucosyltransferase (Gtf) and fructosyltransferase (Ftf). Gtf and Ftf transform glucose and fructose to glucan and fructans, respectively (Munro et al., 1991). Some antiplaque agents inhibit the activity of dental plaques by reducing the production of extracellular glucans (Koo et al., 2000) and fructans (Steinberg et al., 2002).

Glucans and fructans comprise primarily a mixture of different linkages, including α-(1→3), α-(1→6), and β-(1→6) glucans (Bowen and Koo, 2011), as well as β-(2→6)-linked fructan (Willcox and Drucker, 1987). As the key fractions of the matrix, EPS provides sites for the formation of metabolizable polysaccharides, cell aggregation microbial colonization (Koo et al., 2010; Xiao and Koo, 2010), and adhesion among different species (Gregoire et al., 2011). The α-(1→3)-linked glucan is presented in insoluble glucans with high concentrations and α-(1→6)-linked glucan is abundant in soluble glucans (Bowen and Koo, 2011). Glucanohydrolases contain mutanase for insoluble glucans and dextranase for soluble glucan (Hayacibara et al., 2004). Mutanases catalyze the hydrolysis of glucosidic linkages and effectively help fight against *Streptococcus mutans* (*S. mutans*) (Thallinger et al., 2013). These abilities of mutanases mainly manifest in the degree of saccharification and dissolution of water-insoluble EPS in *S. mutans*. Dextranase hydrolyzes dextran, which is an acceptor molecule to synthesize soluble glucans (Xiao et al., 2012). Similar effects have been observed for dispersin B, which hydrolyzes β-(1→6)-glucans (Kaplan et al., 2004). However, breaking one of the linkages in EPS monomers is not sufficient to degrade the biofilm completely. Ren et al. (2019) found that a combination of dextranase and mutanase can synergistically degrade different glycosyl linkages in a biofilm more efficiently.

Moreover, several phenols (including eugenol, catechins, quercetins, and sylvestris) showed similar functions as mutanase or dextranase. Eugenol inhibits both insoluble and soluble glucan activities of *Streptococcus sobrinus* (*S. sobrinus*) considerably (Li et al., 2012). Burt et al., reported that eugenol exhibits significant activity against the biofilm of *Candida albicans* (*C. albicans*) (Burt, 2004). *C. albicans* is one of the major etiological agents in early childhood caries (ECC), which may enhance the virulence of *S. sobrinus* and *S. mutans* as well (Wan et al., 2021). Meanwhile, eugenol presents low cytotoxicity and hemolytic activity. Catechins and quercetins interfere with both insoluble and soluble glucan activities (Zeng et al., 2019) by interacting with Gtf in *S. mutans* (Nakahara et al., 1993). This anti-Gtf action is also associated with sylvestris, which affects the quality of glucans formed by inhibiting GtfB activity. Ribeiro et al. (2019) reported that FLO/SC, PAC/CE, and PRE/SP extracts remove a significant amount of *S. mutans* biofilms, probably because of a decrease in the biomass of glucans produced by GtfB.

Although enzymes that degrade EPS can be used as moderate anti-biofilms agents (Otsuka et al., 2015), their applications alone have not been tested clinically due to their limited antimicrobial activity (Balakrishnan et al., 2000). Promising antibacterial activity in plant species has been noted. Piceatannol could be acted as an inhibitor of *gtfC*, which shares the same space as acarbose (Ito et al., 2011). Due to its specificity for the *S. mutans* Gtf, piceatannol interacted specifically with the adhesion of *S. mutans* biofilms and did not influence cell viability (Nijampatnam et al., 2018). Similarly, osteopontin exhibited an apparent selectivity toward *Streptococcus mitis* SK24 biofilms instead of the planktonic cells by changing the hydrophobicity of the biofilm surface (Schlafer et al., 2012).

Extracellular enzymes can successfully weaken the structure of the biofilms by targeting glucans, fructans, and their different linkages in EPS. Furthermore, a synergistic approach that combines antimicrobial agents with EPS matrix-degrading enzymes can potentially increase the effect of biofilm disruption and prevent dental caries.

#### Deoxyribonuclease

In recent years, environmental DNA (eDNA) has attracted much attention as a component of the matrix of cariogenic biofilms (Pedraza et al., 2017; Tawakoli et al., 2017). There are multiple functions of eDNA in biofilm formation, such as establishing the basis for initial bacterial adhesion and mediating subsequent attachment (Das et al., 2011). In addition, eDNA facilitates the transmission of genetic information among oral biofilms (Roberts and Kreth, 2014). It can also be a source of nutrients, including phosphate, carbon, and fixed nitrogen for oral bacteria (Liu et al., 2018b).

Zhang et al. (2020) designed a type of helical peptide, which could interact with eDNA to induce dispersion of the *S. mutans* biofilm. Several studies have used DNase to cleave eDNA. Endogenous DNase encoded by *deoC* can significantly decrease the biofilm biomass and regulate the dispersion of the *S. mutans* biofilm (Liu et al., 2017). Although DNase barely decreases the viability of planktonic *C. albicans* and *S. mutans*, the human recombinant DNase I can significantly enhance the eradication of dual-species biofilm during its initial stages (Guo et al., 2021). DNase can enhance the susceptibility of antimicrobial agents and their antibiofilm activities by cleaving eDNA.

#### Proteinases and proteins inhibitor

Besides EPS and eDNA, proteins also act as a scaffold to protect the community. Bacteria produce multiple proteins to enhance bacterial adhesion. Surface adhesion proteins, including glucan-binding proteins (GbpA, GbpB, GbpC, and GbpD) and streptococcal protein antigen P (SpaP), can facilitate sucrose-dependent attachment of matrix glucans, salivary agglutinin, and bacteria (Biswas and Biswas, 2005). For instance, Gbp in *S. mutans* can mediate the adhesion of *S. mutans* and promote the functions of the viscoelastic structure (Matsumoto-Nakano, 2018). Proteinase acting on the Gbp proteins exhibits an anti-Gtf effect, which leads to a reduction in the volume of EPS or even bacterial biomass. Proteinase K could affect the biofilm infrastructure of *S. mutans* and *Streptococcus oralis (S. oralis)* by removing most of the extracellular proteins (Karygianni et al., 2020). Flavonoids have a similar function as proteinase. They not only interact with extracellular and soluble proteins on the bacterial surface but also inhibit the activity of Gtfs (Koo et al., 2003).

In sum, endogenous and exogenous nucleases for eDNA and proteinase for surface adhesion proteins may effectively promote the dispersion of cariogenic biofilm.

### Electrostatic interactions agents

The electrostatic interactions involved in the bonding of the biofilm matrix could be affected by hydrophilic agents (Venault et al., 2014), surfactants (Wang et al., 2021), and metal chelators (Roman et al., 2014). Such electrostatic interaction agents have been found to destabilize the biofilm matrix and facilitate biofilm separation (Xavier et al., 2005). However, there are a relatively small amount of studies about electrostatic interactions involved in the bonding of the cariogenic biofilm matrix.

Furthermore, electrostatic interactions exist among anionic metabolites and anionic components on the bacterial surface. Postollec et al. (2003) reported that static electricity affects bacterial adhesion and aggregation via isothermal reaction calorimetry. For instance, the static electricity of polypyrrole affects the positively charged cross-bridging. Most surfaces of the biofilm are negatively charged, and polypyrrole also binds to negatively charged amino acids. Enhancing electrostatic interactions may promote the physical removal of bacteria from the tooth surface by facilitating the biofilm to remain intact and by inhibiting cell separation from long chains. It has been shown that aspartic acid^451^ is a part of the active site that controls the catalytic activity in Gtfs in response to sucrose binding, i.e., the DSIRVDAVD (residues 446–454) (Mooser et al., 1991). High concentrations of polypyrrole can absorb Gtf-I and Gtf-SI and block the action of Gtfs (Kato et al., 1992). Through the electrostatic interactions with *S. mutans*, the polypyrrole structure physically inhibits the formation and colonization of the biofilm. It can also promote the physical removal of the biofilm from the tooth surface by enhancing electrostatic adsorption aggregation (Senpuku et al., 2019).

Either synchronous modification of antimicrobial polyethylene glycol (PEG) or pH-activated charge conversion with cationic peptides has recently emerged as effective approaches to target negatively charged sites (Tian et al., 2020). In this manner, the micelle structures enhanced penetration and self-regulation by anchoring to the targeted biofilm.

Such specific electrostatic interaction agents can facilitate cariogenic biofilm removal by promoting concentrations of effective constituent and affecting the Gtfs.

### Oxygen radicals

During metabolism, endogenous H_2_O_2_ is produced by natural bacteria. The neighboring streptococci in oral micro-ecology, such as *Streptococcus gordonii* (*S. gordonii*)*, Streptococcus sanguis*, and *Streptococcus oligofermentans,* can impact the pathogenesis of *S. mutans* via self-produced H_2_O_2_ (Kreth et al., 2009). H_2_O_2_ generates free radicals, which not only degrade EPS but also promote the physical removal of biofilms by oxidative cleavage (Noyori et al., 2003). Although *S. mutans* is sensitive to oxidative stress (Liu et al., 2018b), the inhibitory effect of *S. gordonii* through H_2_O_2_ is far from adequate (Tanzer et al., 2012).

Exogenous application of H_2_O_2_ is common in household and clinical disinfection. It has little toxicity even at concentrations as high as 10% of effective concentration. The high peroxidase-like catalytic activity of metals or metal oxides under acidic pH has led to an increased interest in their biomedical application. Silver (Metin-Gursoy et al., 2017) and zinc oxide nanoparticles (Hernandez-Sierra et al., 2008), as well as iron oxide nanozymes (Cormode et al., 2018), have been reported to have potent antibiofilm nature. For instance, iron oxide nanozymes in acidic environments have the similar activity as peroxidase. They disrupts the constituents of the biofilm matrix and kill *S. mutans* (Liu et al., 2018a). Dextran-coated iron oxide nanoparticles (Dex-NZM) can degrade EPS at an acidic pH (Naha et al., 2019). Furthermore, the combination of iron oxide nanozymes and H_2_O_2_-generating bacteria improves the overall cleansing effect (Wang et al., 2020). Gao et al. (2016) synthesized catalytic nanoparticles (CAT-NP) to degrade insoluble glucans by the generation of free radicals from H_2_O_2_ in pathogenic acidic biofilms.

Photosensitizer (PS) can also activate molecular oxygen radicals and produce reactive oxygen species (ROS) (Cieplik et al., 2018). Through an oxidative burst, the PS compounds cause bacterial death and biofilm dispersion (de Souza et al., 2020; Martins Antunes de Melo et al., 2021). This method provides a robust direct ablation without drug resistance (Zhao et al., 2019a). Methylene blue (MB) caused a significant reduction in *S. mutans* biofilms, allowing the prospect of eliminating bacterial infections in deep carious lesions (Legenova et al., 2020). Fotoenticine (FTC) is a new derivative of chlorin e-6, which showed significant photodynamic effects against cariogenic bacteria, including *S. mutans* that was isolated from patients with dental caries (Terra Garcia et al., 2018). Due to the high carbohydrate content, the *S. mutans* biofilms exhibited greater absorption to PS than fungal cells, which might be the reason for the susceptibility of *S. mutans* (Sharma et al., 2011). Even in a complex polymicrobial biofilm, *S. mutans* are more susceptible to FTC-mediated photodynamic therapy (Garcia et al., 2021).

H_2_O_2_ and nanoparticles with the peroxidase-like activity present an ideal antibiofilm strategy by generating free radicals for the elimination of oral biofilms.

## Modulation of signaling pathways

Instead of targeting the biofilm matrix, small molecules have been used to influence signaling systems by disaggregating bacteria (Ren et al., 2016; Fleming and Rumbaugh, 2017; Snarr et al., 2017). Due to its unique patterns of gene expression and protein production in each developmental stage of biofilms, bacterial signaling systems can minimize the impact on normal bacterial flora and prevent dental plaque infectious diseases (Lamont et al., 2018).

### Quorum sensing

Quorum sensing (QS) is a microbial communication response in the entire cell population and has a significant impact on the biofilm life cycle (Li and Tian, 2012). QS is a typical microbial communication mode that enables bacteria to display cooperative group mechanistic behavior, which controls the expression of genes to virulence factors, biofilm dispersion, biofilm activity, and secondary metabolism (Li et al., 2001). Therefore, inhibition of the QS pathway would be a potential strategy for attenuating bacterial virulence.

#### The comCDE system

The comCDE system and the agglutinin-like sequence (Als) family are important in QS. The comCDE system responds to environmental signals, such as acid, and mediates pheromone competence stimulating peptide (CSP) activity (Lemos and Burne, 2008). High concentrations of CSP, which is a QS molecule in streptococci, may reduce biofilms and elongate the cells (Qi et al., 2005). Cvitkovitch et al., synthesized an analog of CSP (KBI-3221), which specifically targeted the QS pathway and decreased biofilms in various streptococcus biofilm dispersal (LoVetri and Madhyastha, 2010). Carolacton triggered the death of *S. mutans* by interfering with the comCDE system, and ComX in a growth-dependent way (Kunze et al., 2010).

Curcumin could downregulate the expression of the comCDE system (comC, comD, and comE) (Li et al., 2019) to inhibit QS (Li et al., 2018) and alter the EPS production (Falsetta et al., 2014). Hoyer et al., indicated that the expression of the Als family in *C. albicans*, which controls adhesion and aggregation, is suppressed by curcumin (Hoyer and Cota, 2016).

#### The *LuxS* system

In *S. oralis*, *S. gordonii*, and *S. mutans*, sulfated vizantin (Viz-S) reduces the expression of luxS and the downstream pathway of AI-2. With the deletion of the *luxS* gene, *gtfB* and *gtfC* genes are upregulated, which markedly reduces biofilm formation (Yoshida et al., 2005). Activation of the *luxS* gene downregulates the expression of *gtfG* in *S. gordoni (*Mcnab et al., 2003*)*. AI-2 was also found in the inner cellular matrix of *S. mutans* and *S. sobrinus*. AI-2 inhibits the expression of *gbpC* and *dblB*, and induces the production of dextran-dependent aggregation (DDAG) (Lee et al., 2015).

Downregulation of the *luxS* gene alters biofilm structure in *S. oralis* and *S. gordonii* resulting in dispersion (Cuadra-Saenz et al., 2012).

#### Others

There has been increased interest in the QS system for the development of Chinese traditional medicine in recent years. Zingiber officinale reduces the expression of the entire set of *S. mutans* virulent genes and genes related to the biofilm life cycle, including *comDE* (for part of the QS cascade), relA (for oxidative stress and acid tolerance mechanisms) (Liu et al., 2011), *brpA* (for biofilm development and maturity), and *gtfC* (for the synthesis of glucans). The repression of these genes, especially their inhibition through the QS system, would attenuate their internal communication systems (Hasan et al., 2015). Cannabigerol also exerted an anti-bacterial effect against *S. mutans* (Karas et al., 2020; Aqawi et al., 2021). Cannabigerol suppressed the expressions of *gbpB* (for growth essential), *vicR (*for cell wall derivation and biofilm formation) (Lei et al., 2018), *brpA* (Wen et al., 2018), and *wapA* (for cell aggregation and biofilm architecture) (Zhu et al., 2006), with a concomitant increase in *spaA* (for binding *S. mutans* to tooth surfaces) expression and activity (Yang et al., 2019). Taken together, the above findings show that affecting the QS pathway can alter various gene expressions and attenuate the internal communication system, which may lead to biofilm disruption.

### The *Gtf* gene family

All the QS pathways mentioned above involving the *Gtf* gene family. As we mentioned before, Gtfs maintain the integrity of the biofilm (Klein et al., 2015). The *Gtf* gene family, which encodes all Gtfs in *S. mutans*, directly responds to glucan matrix formation (Lei et al., 2015) and is regulated by the *rnc* gene. Increased expression of the *rnc* gene down-regulates *vicRKX* by posttranscriptional repression, followed by the promotion of the expression of *gtfB* and *gtfC* genes (Stipp et al., 2013; Mao et al., 2016). Therefore, the *rnc* gene could be responsible for decreasing the EPS (Mao et al., 2018).

Mao et al. (2021) reported that graphene oxide with Cu nanocomposites (GOCuNPs) can the antibacterial effects by decreasing the expression of the *rnc* gene. The regulatory role of graphene oxide with Ag nanocomposites has been reported to be the same as GOCuNPs. They can alter the QS gene expressions of *S. mutans* and the biological process of adherence (Kulshrestha et al., 2017). GOCuNPs can also regulate the expression of the Cop family, including *CopA* (for P1-ATPase copper export), *CopY* (for negative DNA-binding repression), and *CopZ (*for copper chaperone) (Garcia et al., 2016). Cu is consistent with the effect of GOCuNPs in transcriptional repression of Gtfs by inhibiting the expression of the Cop family (Singh et al., 2015).

Besides being regulated by the QS system (Viszwapriya et al., 2017), WIG-synthesizing *Gtf* genes promoted caries in *Streptococcus* species (Xu et al., 2018). Therefore, *Gtf* genes family plays an important role in biofilm dispersion.

### Cue sensing

In addition to QS, cue sensing also plays a key role in bacterial communication. Cue sensing and its signal transmission eventually lead to the downregulation of the cyclic di-guanosine monophosphate (c-di-GMP). c-di-GMP is an intracellular secondary messenger for signal transduction. c-di-GMP-based regulatory systems are involved in diverse aspects of each stage of biofilm development, including biofilm dispersion (Rumbaugh and Sauer, 2020).

Peng demonstrated that *S. mutans* modulates the production of EPS and biofilm formation by regulating c-di-AMP levels (Peng et al., 2016). The *gcp* gene in *S. mutans* encodes AAN59731, which is a conserved hypothetical protein, which acts as a diadenylate cyclase (Yan et al., 2010). It was reported that downregulation of cdaA decreases the production of diadenylate cyclase and the levels of c-di-AMP, resulting in reduced EPS content and increased sensitivity to H_2_O_2_ (Cheng et al., 2016). Due to a reduction in c-di-GMP levels, the expression of matrix-degrading enzymes increases, resulting in matrix dispersal (Romling et al., 2013; Srivastava et al., 2013). Therefore, a decrease in the levels of c-di-GMP induces biofilm dispersion to planktonic mode, while an increase in intracellular c-di-GMP levels fosters it to a sessile mode (Hengge, 2009).

### *YidC* family

The deletion of *YidC* in *S. aureus* inhibited biofilm formation and attenuated virulence. In *Escherichia coli*, *YidC* mutations were lethal (Samuelson et al., 2000). Although with phenotypic differences, mutants of either *YidC1* or *YidC2* still reduce virulence in *S. mutans* (Palmer et al., 2012; Crowley and Brady, 2016). Particularly, *YidC2* has recently been identified to have the capability of folding plasminogen-binding protein (PBPs) and secreting enzymatic activities. Therefore, deletion of *YidC2* causes significant alterations not only in cell physiology properties and division, but also in the EPS matrix assembly and mechanical stability associated with dental caries (Palmer et al., 2019).

## Biological regulation of microbial homeostasis

### Bacteriophages

Bacteriophages are viruses that invade bacteria with high strain specificity and low toxicity (Chan and Abedon, 2015). When bacteriophages infect bacteria, they induce EPS depolymerization and lysis, which degrades the biofilm matrix and impairs cell wall integrity (Azeredo and Sutherland, 2008). After accessing the biofilm, bacteriophages disrupt key metabolic processes, such as the QS system, and even affect the regulation of the eDNA release, which induce bacterial lysis (Rehman et al., 2019). Bacteriophages are good candidates for genetic engineering. They can co-evolve with the bacterial host to resist the antibiotic (Khalifa et al., 2016). Dalmasso et al. (2015) isolated phage, ɸAPCM01, successfully. ɸAPCM01 is a *S. mutans* bacteriophage that inhibits the growth of *S. mutans* and efficiently destroys its biofilms. SMHBZ8 is also a *S. mutans* bacteriophage that is isolated from salivary samples and it has similar antimicrobial properties as ɸAPCM01 (Ben-Zaken et al., 2021). Overall, by invading bacteria, bacteriophages offer a broad prospect to be used as a novel biotherapy.

### Probiotics

Probiotics treat oral infections by developing a symbiotic or reciprocal relationship with the host (Roberts and Darveau, 2015). They can prolong the therapeutic efficacy by niche occupation and prevent recolonization of the pathogenic bacteria. An ecological approach to caries treatment is to modulate and maintain the beneficial properties of the indigenous oral microflora.

There are already various commercial mouthwash and lozenges that are supplemented with probiotic bacteria, such as PerioBalance®, KForce Breath Guard®, and ProBiora3® (Yao and Fine, 2014). *Streptococcus salivarius* (*S. salivarius*) K12 and *Lactobacillus rhamnosus* GG are probiotic formulations for oral health (Caglar et al., 2005). Aggregation of *S. mutans* can cover up their surficial sites, rendering them unavailable for drug binding (di Cologna et al., 2021). Co-aggregation of *Lactobacillus paracasei* DSMZ16671 and *S. mutans* exposes these sites and removes *S. mutans* without disruption of other oral commensal species (Lang et al., 2010). Besides, *lactococcus* such as *Lactococcus lactis* produces nisin and disrupts oral pathogenic biofilms (Radaic et al., 2020). Therefore, by maintaining a healthy balance, probiotic bacteria and their metabolite can inhibit the process of biofilm development and preserve the beneficial properties of the oral microflora.

## Dispersion promotion with nanovehicles

Due to the particularity of tooth anatomical structure, improper treatment for biofilm removal may expose pulp tissue or adjacent soft tissue (Schwendicke et al., 2018). Potent antibiotics, such as CHX, are significantly cytotoxic with side effects, including discoloration or nerve damage due to pulp exposure (Nemezio et al., 2017). Furthermore, bacteria that may survive in the inner layer or the unintentional removal of tissues can weaken the tooth structure and even cause toothaches (Orhan et al., 2010). However, we can still take advantage of biofilm infiltration and intramembrane transport of drug delivery nanotechnology (Zhou et al., 2016). Polymer micelles (Zhao et al., 2019b), vesicles (Xi et al., 2019), and liposomes (Benoit et al., 2019) have been proven to have great potential for drug delivery. These nanocarriers are ideal materials with high surface area and specific catalytic and magnetic properties for use in nanomedicine (Ramos et al., 2017). Nanocarriers loaded with antimicrobials have displayed unique characteristics, including targeted bacterial enzyme decomposition of micellar carriers (Li et al., 2016) and enhanced infiltration or accumulation (Landis et al., 2017).

### Common nanocarrier

Chitosan is a common nano-carrier, which can interact with both biofilm bacteria and enamel (Li et al., 2013). Chitosan, as a bio-adhesive polymer, can improve the adherence of its contents and interfere with the adhesion of biofilm bacteria (Aliasghari et al., 2016). Covarrubias et al. (2018) demonstrated that Cu coating inside chitosan (CuChNP) improves the adherence of Cu to *S. mutans* and the tooth surface. CuO-chitosan hybrid structure, silver nanoparticles containing lactose-modified chitosan (Chitlac-nAg) (Ionescu et al., 2015), poloxamer 407 formulations, capped lysozyme, and lactoferrin nanoparticles are known to reduce *S. mutans* biofilm burden (Tonguc-Altin et al., 2015). Nanocarriers, such as chitosan, can increase adherence or aggregation of the active ingredient to improve biofilm dispersion.

### Target nanocarriers

One of the most important features of cariogenic biofilm microenvironments is their acidic nature. Once inside a biofilm, pH-responsive nanocarriers would expedite the release of antimicrobials through degradation of their biodegradable linkages. Zhao et al. (2019b) designed a pH-responsive detachable PEG shell that infiltrated the oral biofilms and embedded itself in the interlayer of the nanoplatforms through dynamic borate linkages. In the weakly acidic micro-ecological environments (pH 6.5), the linkages shed their PEG coating. The pH-responsive nanoparticles are capable of readily binding to EPS and reinforcing its penetration, which leads to enhanced drug anchorage followed by “on-site” drug release. Collectively, it can be a feasible strategy for the treatment of dental caries.

Specifically-targeted antimicrobial peptides (STAMPs) ensure targeted delivery to specific species in a mixed-species environment. Eckert et al. (2006) designed a STAMP molecule by combining a species-specific targeting peptide and a non-specific killing peptide. This STAMP bound specifically to *S. mutans* and eliminated it effectively while maintaining a healthy biofilm. It also showed considerable protective effects with the competitiveness of healthy normal flora against *S. mutans* colonization (Li et al., 2010).

Dextranomer (DMs) has a similar targeted delivery function as STAMPs with different principles. DMs exhibit a specific affinity for pathogenic oral streptococci, while causing limited disturbance to healthy biofilms. The affinity between DMs and oral streptococci may increase depending on the presence of sucrose. DMs with antimicrobial cargo not only protect healthy bacteria, but also improve bacterial aggregation of selectively adhered bacteria (Mashburn-Warren et al., 2017). Targeting a particular microbial species or a specific kind of pathogen can help maintain microbial homeostasis, and thus, and better eliminate pathogens significantly.

### Multifunctional nanocarriers

Nanotechnology-based therapeutic modalities provide many versatile strategies to coordinate biofilm infiltration and bacterial anchoring functions.

To combine pH-adaptive nanocarriers and positive surface charge therapy, Benoit et al., developed p(DMAEMA)-b-p(DMAMEA-co-BMA-co-PAA) nanocarriers, which offer outstanding adhesion effect and can target negatively charged tooth matrix or biofilm components for drug accumulation in cariogenic biofilms (Horev et al., 2015). Furthermore, most of the cariogenic *S. mutans* are characterized by esterase activity, which degrades the ester-linkage of PAE (Hansel et al., 1998). Under acidic conditions, PAE is exposed, and can penetrate and accumulate in the biofilm. It also targets negatively charged bacterial cell surfaces with its positive charge (Liu et al., 2016). Combining the function of stealthy penetration with low pH and electrostatic attraction allows accumulation in biofilms. Therefore, PEG-PAE micelles significantly increase the efficacy of Triclosan (Wang et al., 2016). Such properties thwart dental caries by the enrichment of local drugs. The high drug bioavailability impacts overall biofilm dispersion, allowing bacterial retention at the infection site, which is a highly promising strategy for efficient bacteria killing.

## Conclusion

In this review, we summarized the applications and mechanisms of the strategies for dispersion of cariogenic biofilms. Most of the studies that we have discussed focus on mono species. However, the real cariogenic biofilms comprise various acidogenic and aciduric microorganisms, including *S. mutans*, *S. sobrinus*, *Lactobacillus reuteri*, and even fungi (Pires et al., 2019). In addition, the interaction between pathogenic species and salivary components can help bacterial species adapt to environmental stress, while aiding in the bacterial evolution of cariogenic biofilms. This phenomenon is referred to as horizontal gene transfer (HGT) (Kim et al., 2017). HGT is the main means for species to exchange metabolites and generate resistance (Lobo et al., 2019). Therefore, it is necessary to expand research on dual-species biofilms and biofilms with mixed pathogens.

Although much research has addressed bacterial biofilms, experimental conditions vary from one study to another. The oral hygiene of patients is also dependent on individual cleaning habits and orthodontic appliances used. There are novel research models that mimic the oral environment. To close the knowledge gap between ideal experimental conditions and the actual oral environment, more suitable experimental models and *in vivo,* mechanistic models are needed. Such research will play an important role in facilitating practical clinical applications. Furthermore, such therapeutic strategies can potentially be extended to other pathological conditions, such as periodontitis (Natan and Banin, 2017; Sun et al., 2021), and microbial communities. Useful strategies are by no means limited to one condition. Further research that aims to improve available strategies can shift their time, the proportion of medication applied, and dependence on auxiliary medical equipment, such as irradiation.

Many Chinese medicine ingredients comprise natural products that can contribute to overcoming the problem of chemical agents, including narrow specificity, slow action, expensive manufacturing, and drug purification for biomedical applications (Hannig et al., 2010). Focusing on strategies that can achieve biofilm dispersion to a certain degree can help preserve a balanced oral microbiome, and thus, can aid in preventing drug-resistant bacteria. It is worth noting that some of the strategies should be used together with antimicrobials to maximize biofilm dispersion. Based on the review of numerous relevant studies, we can improve therapeutic approaches by combining strategies instead of monotherapies (Xiao et al., 2018).

## Author contributions

RC wrote the manuscript. CL designed this project and wrote the manuscript. MD designed this project. All authors contributed to the article and approved the submitted version.

## Funding

This study was funded by grants from the National Natural Science Foundation of China (grant nos. 81201260 and 81771084).

## Conflict of interest

The authors declare that the research was conducted in the absence of any commercial or financial relationships that could be construed as a potential conflict of interest.

## Publisher’s note

All claims expressed in this article are solely those of the authors and do not necessarily represent those of their affiliated organizations, or those of the publisher, the editors and the reviewers. Any product that may be evaluated in this article, or claim that may be made by its manufacturer, is not guaranteed or endorsed by the publisher.
